# The protective effect of *Blautia coccoides* in secondary injury of intracerebral hemorrhage

**DOI:** 10.3389/fmicb.2025.1616222

**Published:** 2025-09-02

**Authors:** Xian Shao, Matao Zheng, Chenglong Ye, Jiafeng Lu, Mengyun Li, Kuiwei Su, Hui Lin, Lingyan He, Xuchen Qi, Jianli Wang

**Affiliations:** ^1^Department of Medical Research Center, Shaoxing People’s Hospital, Zhejiang University Shaoxing Hospital, Shaoxing, Zhejiang, China; ^2^Second Clinical Medical College, Zhejiang Chinese Medical University, Hangzhou, Zhejiang, China; ^3^Department of Neurosurgery, Shaoxing People’s Hospital, Zhejiang University Shaoxing Hospital, Shaoxing, Zhejiang, China; ^4^Department of Stomatology, Shaoxing People’s Hospital, Zhejiang University Shaoxing Hospital, Shaoxing, Zhejiang, China; ^5^Department of Traditional Chinese Medicine, Shaoxing People’s Hospital, Zhejiang University Shaoxing Hospital, Shaoxing, Zhejiang, China; ^6^Department of Neurosurgery, Sir Run Run Shaw Hospital, Zhejiang University School of Medicine, Hangzhou, Zhejiang, China

**Keywords:** intracerebral hemorrhage, gut microbiota, *Blautia coccoides*, neuroprotection, neuroinflammation

## Abstract

Intracerebral hemorrhage (ICH) is a severe stroke with high rates of disability and mortality. Emerging evidence suggests a link between neurological disorders and gut microbiota dysbiosis, though the underlying mechanisms remain unclear. To investigate the role of gut microbiota in ICH, we conducted metagenomic analysis of fecal samples from 35 healthy individuals and 36 patients with ICH, including 29 survivors and seven deceased patients. Metagenomic analysis revealed decreased gut microbiota diversity in patients with ICH, with *Blautia* genus identified as potential biomarkers. We also established an ICH mouse model via stereotactic autologous blood injection to assess the therapeutic potential of *Blautia* genus. *Blautia coccoides* (BC), a representative strain, improved neurofunctional outcomes in ICH mice, reduced tissue damage and neuronal apoptosis, and decreased glial cell activation markers (GFAP and Iba1). BC treatment also lowered the serum levels of pro-inflammatory cytokines (TNF-α, IL-6, and IL-1β) and partially restored gut microbial homeostasis. These findings suggest that BC plays a crucial role in ICH progression and may serve as a potential therapeutic agent by modulating gut microbiota. Further research and clinical trials are necessary to validate its efficacy and safety in humans.

## 1 Introduction

Intracerebral hemorrhage (ICH), often referred to as spontaneous ICH, is a medical condition characterized by bleeding within the brain resulting from the rupture of blood vessels. ICH is among the most prevalent and challenging conditions encountered in neurosurgery, associated with a high mortality rate and a poor prognosis. It is a leading cause of mortality and disability globally. Mortality rates range from 27% to 50% within the first month following an ICH event, approximately 50% at 12 months, around 70% at 5 years, and exceed 80% at 10 years post-onset. Approximately 80% of ICH survivors experience some form of disability 6 months after the event, with only 14% – 36% achieving functional independence at 1 year (Magid-Bernstein et al., 2022; Xu L. et al., 2024). The primary etiological factors of ICH include aging, hypertension, and cerebral amyloid angiopathy, while secondary causes comprise vascular abnormalities detectable through imaging, hemorrhagic tumors, and related conditions (Magid-Bernstein et al., 2022; Ma et al., 2023; Huang et al., 2024). Acute brain injuries caused by ICH can be divided into primary and secondary types. Primary injury includes mechanical damage caused by primary bleeding or hematoma expansion, leading to congestion (Wan et al., 2023). Secondary injury is a pathological process that develops over time based on primary injury and is characterized by progressive worsening of the level of brain damage (Chen et al., 2021). Primary causes of secondary injury include neuroinflammation, neuronal apoptosis, excitotoxicity, and oxidative stress. Secondary injury can develop over hours, days, or even weeks; Secondary injury substantially elevates risks of mortality and long-term disability, thereby worsening overall patient prognosis. Therefore, it is essential to intervene and treat secondary injuries following ICH (Cong et al., 2024; Loan et al., 2022). Currently, the primary goal of surgical treatment is to promptly evacuate the hematoma and relieve brain compression. However, this approach did not improve neurological function in patients with ICH (Al-Kawaz et al., 2020). Therefore, there is an urgent need for the development of novel therapeutic strategies.

In recent years, numerous studies have confirmed the correlation between gut microbiota and brain function, with dysbiosis of the gut microbiota affecting the occurrence and development of central nervous system diseases, including alzheimer’s disease, parkinson’s disease, and stroke (both ischemic and hemorrhagic) (Doroszkiewicz et al., 2021; Socała et al., 2021). Studies indicate that during both the acute and chronic phases, ICH mice exhibit significantly reduced gut microbiota diversity and richness. Specifically, significant reductions were observed in *Firmicutes*, *Ruminococcaceae* (Li et al., 2023). The impact of gut microbiota on brain function following ICH is mainly manifested in the following aspects: (1) The gut microbiota can alter intestinal defense function and gut permeability, influencing the prognosis of cerebral hemorrhage. (2) Gut microbiota can influence risk factors related to the progression of ICH. (3) The gut microbiota participates in the pathological process of ICH by affecting the expression of gastrointestinal peptides (Wen et al., 2024; Klepinowski et al., 2023). Current studies have documented gut dysbiosis in ICH patients, demonstrating dynamic alterations throughout disease progression. Intestinal homeostasis shows strong linkages to neurological recovery, with families such as *Lachnospiraceae* and *Bacteroidaceae* exhibit reduced abundance during ICH, suggesting their potential protective role in ICH prognosis. Conversely, *Enterococcus* demonstrates significant enrichment throughout ICH progression, representing a pathogenic risk factor for ICH (Wang et al., 2024; Luo et al., 2022).

The *Blautia* genus is a type of anaerobic bacterium with probiotic characteristics belonging to the Firmicutes phylum, *Lachnospiraceae* family, and is found in mammalian feces and intestines. *Blautia* genus are strictly anaerobic, non-motile, and important core genera in the mammalian gut with 20 valid published species (Liu et al., 2021). *Blautia coccoides* (BC) is a representative strain of the *Blautia* genus and is known for its significant anti-inflammatory effects (Weiss et al., 2023).

In this study, we systematically investigated the changes in the gut microbiota at the clinical level during the development of ICH. The genus *Blautia* may represent key microbial contributors to the pathological progression of ICH. We identified the key bacterial *Blautia* genus and validated its therapeutic effects against secondary injury in an animal model of ICH. This study provides a new direction to treat ICH-induced secondary injuries.

## 2 Materials and methods

### 2.1 Patients and sample collection

From January 2024 to September 2024, following the guidelines of the Ministry of Health, 36 patients diagnosed with hypertensive ICH were recruited at Shaoxing People’s Hospital, of whom seven patients were deceased. Data on clinical manifestations were retrieved, and patients with malignant tumors, diabetes, neurological disorders, or other metabolic diseases were excluded based on brain CT scans and NSS scores. Fecal samples during the recovery period were collected from surviving patients, designated as the ICH-S group, whereas fecal samples from the 6 h before death were collected from deceased patients, designated as the ICH-D group. In addition, 35 healthy individuals living in the same area as the patients were recruited, and their specimens were stored in the Human Genetic Resource Sample Repository of the Shaoxing People’s Hospital. The demographic profiles, clinical data, and sampling time of all enrolled participants are comprehensively detailed in Supplementary Table 1. This study was approved by the Ethics Committee of Shaoxing People’s Hospital and written informed consent was obtained from each participant (Approval no. IEC-K-AF-016-1.2).

### 2.2 Animal model

The 8-week-old male C57BL/6J mice were purchased from Qizhen Co. Ltd. (Hangzhou, China). The mice were maintained in a standard environment (temperature, 22 ± 2°C; humidity, 50% ± 10%) under specific pathogen-free conditions. All animal experiments were approved by the Animal Ethics Committee of Shaoxing People’s Hospital (Approval no. 2023Z034).

### 2.3 ICH model

The mouse ICH model was established using autologous blood stereotactic injection (Autologous blood was collected aseptically from the tail vein), as previously described (Cheng et al., 2024). Briefly, mice were fasted for 12 h, mice were anesthetized with 2% sevoflurane (H20070172, Hengrui, Shanghai, China) gas with 0.4 mL/min at 4 L/min fresh gas flow using small animal gas anesthesia machine (R500IP; RWD Life Technology Co., China) during the surgical procedure, placed in a prone position, and fixed on a stereotactic apparatus (69100; RWD Life Technology Co., China). A midline incision was made along the sagittal suture to expose mouse fontanelles. Autologous blood (30 μL) was slowly injected into the right basal ganglia area (3.0 mm lateral to the fontanelle, 1 mm posterior, and 4.0 mm below the skull) within 20 min. To prevent reflux, the needle was withdrawn in two steps at 5-min interval, after which the needle was left in place for 5 min before suturing the scalp. A temperature-controlled system at 37°C was used both intraoperatively and postoperatively. The sham group underwent the same procedure but without an autologous blood injection.

### 2.4 BC culture

*Blautia coccoides* strain was obtained from the American Type Culture Collection (ATCC, catalog no. 29236) and cultured in chopped meat carbohydrate broth (CMC, Hopebio, China) at 37°C under anaerobic conditions for 48 h. The BC density was determined by measuring the optical density at 600 nm (OD_600_). The bacteria were centrifuged at 3000 rpm for 15 min at 4°C, followed by resuspension in PBS.

### 2.5 Experimental groups and drug treatment

The mice were randomly divided into three groups: sham, ICH, and BC intervention groups (BC group), with nine mice in each group. One day after ICH modeling, the mice were orally administered either 1 × 10^9^ CFU of BC (BC group) or CMC culture medium (ICH and sham groups) for three consecutive days. Behavioral assessments in ICH mice were performed at 3 and 7 days post-modeling, respectively.

### 2.6 Brain water content measurement

Brain water content was determined using the wet/dry method as described previously (Xu Y. et al., 2024). Briefly, mice were euthanized by decapitation under deep anesthesia, the mouse brain was removed and the right hemisphere was excised and weighed (wet weight). Tissues were dried at 100°C for 48 h and weighed (dry weight). Brain water content was calculated as:

Brain water content = [(wet weight–dry weight)/wet weight] × 100%.

### 2.7 Behavior tests

#### 2.7.1 Modified mouse neurological severity scores (mNSS)

Consistent with previous studies (Li et al., 2024), a mNSS score was used to comprehensively evaluate motor, sensory, and balance functions in the model mice. The severity was determined as follows: 0, without dysfunction; 1–6, mild damage; 7–12, moderate damage; and 13–18, severe damage.

#### 2.7.2 The corner turn test

The corner test apparatus comprises two boards formed at an angle of 30° on the platform. The mice progressed to the 30°corner and then turned either to the left or right. The choice of turning direction was recorded over 10 trials, and the percentage of left turns was reported as the outcome.

#### 2.7.3 The open-field test

The mice were placed in the center of an open-field test chamber (25 × 25 cm dark box, Panlab, RWD Life Technology Co., China) and allowed to freely explore for 3 min. The activity of the mice was recorded using a camera, and the data were analyzed using an open-field behavior analysis system (SMART V3.0, RWD Life Technology Co., China). Movement distance and immobility time of the mice were used to evaluate motor function.

### 2.8 Tissue histology and staining

After being anesthetized deeply, mouse brains were fixed in a 4% paraformaldehyde solution (G1101, Servicebio, China) for fixation and dehydration. After fixation, the tissues were embedded in paraffin and sliced into 4 μm-thick sections (CM1950, Leica, Germany). Quantification and statistical analysis of target regions using the ImageJ software.

#### 2.8.1 Hematoxylin and eosin (H&E) staining

For H&E staining, the procedure was performed following the manufacturer’s protocol of the HE staining kit (C0105S, Beyotime, China). Briefly, paraffin sections were immersed in a hematoxylin solution for 2 min, followed by quick transfer to an eosin solution for 30 s. Stained sections were observed under an optical microscope (DMi1, Leica, Germany).

#### 2.8.2 Nissl staining

For Nissl staining, paraffin sections were immersed in Nissl stain (C0117, Beyotime, China) in an oven at 45°C for 5 min, and the results were observed under an optical microscope (DMi1, Leica, Germany).

#### 2.8.3 Immunofluorescence

For immunofluorescence, paraffin sections were blocked in 2% bovine serum albumin at room temperature for 1 h and incubated with the corresponding primary antibodies (glial fibrillary acidic protein (GFAP), 1:100, 16825-1-AP, Proteintech, China; Ionized calcium-binding adaptor molecule 1 (Iba1), 1:100, 10904-1-AP, Proteintech, China) overnight at 4°C. The following day, sections were incubated with CoraLite488-conjugated Goat Anti-Rabbit IgG (H + L) (SA00013-2; 1:100, Proteintech, China) at room temperature for 2 h. Cells were observed under a confocal microscope (STELLARIS 8, Leica, Germany) in the dark.

#### 2.8.4 TdT-mediated dUTP nick-end labeling (TUNEL) staining

A TUNEL apoptosis detection kit (C1091, Beyotime, China) was used to visually detect apoptotic cells. After routine deparaffinization and hydration, deparaffinized tissue sections were incubated in proteinase K buffer and TUNEL detection solution for 15 and 60 min, respectively. Subsequently, DAPI staining solution was added. Finally, slides were mounted in glycerol. Cells were counted under a microscope (STELLARIS 8, Leica, Germany).

### 2.9 Western blotting

Briefly, the mouse brain tissue was lysed in RIPA buffer (P0038, Beyotime, China) containing 1% protease inhibitors (ST506, Beyotime, China). After denaturation at high temperatures, proteins were separated by SDS-polyacrylamide gel electrophoresis and transferred onto a PVDF membrane (IPVH00010, Millipore, USA). The membrane was incubated with the corresponding primary antibody overnight at 4°C (BAX, 1:1000, 50599-2-Ig, Proteintech, China; Bcl2, 1:1000, 26593-1-AP, Proteintech, China; β-Actin, 1:5000, 66009-1-Ig, Proteintech, China), followed by incubation with a horseradish peroxidase-conjugated secondary antibody (HRP-conjugated Goat Anti-Rabbit IgG (H + L), 1:1000, SA00001-2, Proteintech, China; HRP-conjugated Goat Anti-Mouse IgG (H + L), 1:1000, SA00001-1, Proteintech, China). Protein bands were detected using an ECL chemiluminescence kit (P0018FM, Beyotime, China) with a ChemiDoc Touch Gel Imaging System (Bio-Rad, USA), and the target regions were quantified and analyzed using ImageJ software.

### 2.10 Enzyme-linked immunosorbent assay (ELISA)

To measure IL-6, IL-1β and TNF-α levels, commercial ELISA kits (IL-6: BMS603-2HS, Invitrogen, USA; IL-1β: EK201BEGA, LiankeBio, China; TNF-α: EK201BEGA; LiankeBio, China). The optical density values were measured using a multifunctional microplate reader (TECAN, Switzerland), and the relative concentrations of the samples were calculated from a standard curve.

### 2.11 Metagenomic sequencing

Total DNA was extracted from the fecal samples using a fecal DNA extraction kit (DP328-02, Tiangen, China). Briefly, sequencing libraries were generated using a NEBNext Ultra II DNA Library Prep Kit (New England Biolabs, Ipswich, MA, USA) following the manufacturer’s recommendations. The quality of the constructed DNA libraries was confirmed using a Qubit 2.0 Fluorometer (Thermo Fisher Scientific, Waltham, MA, USA). The libraries were sequenced using a NovaSeq 6000 instrument (Illumina, San Diego, CA, USA). All samples were sequenced with a 150 bp read length to a targeted data size of 3 GB. Data analysis was performed using OmicStudio tools created by LC-BIO Co. Ltd. (Hangzhou, China) at https://www.omicstudio.cn.

### 2.12 16S rRNA sequencing

Total DNA was extracted from the fecal samples using a fecal DNA extraction kit. Variable regions 3 and 4 (V3–V4) of the 16S rRNA gene were amplified by PCR from the extracted and purified genomic DNA using forward (ACTCCTACGGGAGGCAGCA) and reverse (GGACTACHVGGGTWTCTAAT) primers. Multiplex sequencing of amplicons with sample-specific barcodes was performed using the Illumina MiSeq platform (Illumina, San Diego, CA, USA). Paired-end reads were merged, trimmed, filtered, aligned, and clustered by amplicon sequence variants (ASV) using DADA2. The merged sequences were analyzed using the QIIME v.1.9.1 software package (Xie et al., 2023).

### 2.13 Untargeted metabolomics

To extract metabolites from the serum, the samples were vortexed in pre-cooled 80% methanol. The samples were then incubated on ice for 5 min and centrifuged at 15,000 × *g* for 15 min at 4°C. The supernatant was collected and injected into an LC-MS/MS system for analysis. An ultra-high-performance liquid chromatography (UHPLC) system (Thermo Fisher Scientific) and an Orbitrap quadrupole-Orbitrap high-resolution mass spectrometer (HF-X mass spectrometer, Thermo Fisher Scientific) were used for UHPLC-MS/MS analysis. Compound Discoverer 3.1 (CD3.1, Thermo Fisher Scientific) was used to process the raw data files generated by UHPLC-MS/MS, including peak alignment, peak picking, and quantification of various metabolites.

### 2.14 Statistical analysis

All results were visualized using GraphPad Prism 5 (GraphPad Software Inc., San Diego, CA, USA). At least three independent replicates were used to ensure accuracy. Statistical analyses were performed using GraphPad Prism 5. Student’s *t*-test was used to compare two groups, and one-way analysis of variance with Tukey’s or Bonferroni’s multiple comparison test was used to assess the significance in more than two groups. Statistical significance was set at *p* < 0.05 significant. The mean ± standard error of the mean was reported for all data.

## 3 Results

### 3.1 Blautia decreased in the gut of patients with ICH

**FIGURE 1:**
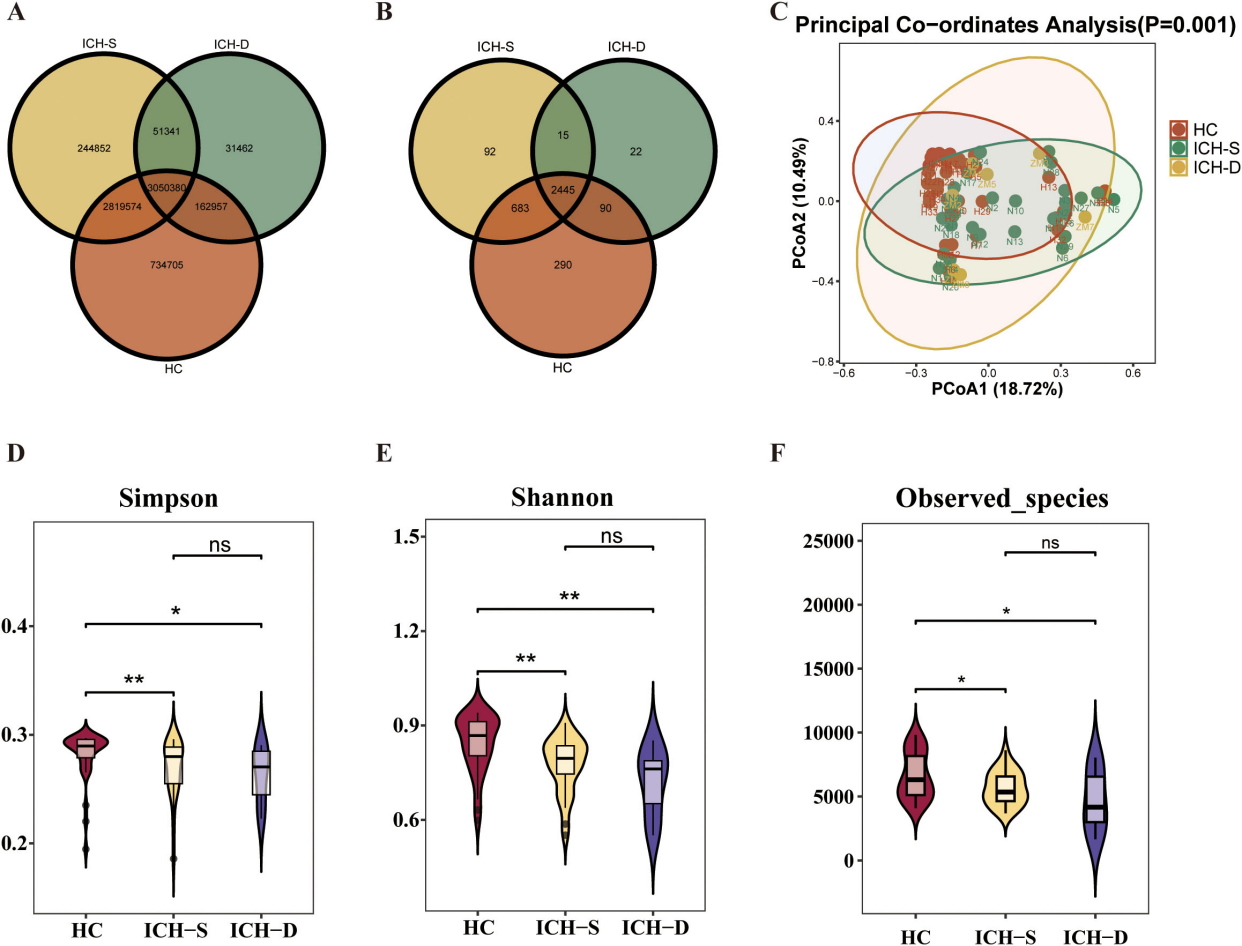
Metagenomic analysis of gut microbiota in patients with ICH. **(A)** Venn diagram of the gut microbiota genome counts in HC, ICH-S, and ICH-D mice. **(B)** Venn diagram of the gut microbiota counts at the genus level in the three groups. **(C)** The β-diversity of the gut microbiota was represented by PCoA analysis of the three groups. **(D–F)** The α-diversity of gut microbiota is represented by Simpson, Shannon, and observed species indices in the three groups. (*p < 0.05, **p < 0.01. HC *n* = 35, ICH-S *n* = 29, ICH-D *n* = 7).

Thirty-six fecal specimens from patients with ICH (survivors in the ICH-S group and non-survivors in the ICH-D group) and 35 healthy controls were collected for metagenomic sequencing. Based on metagenomic gene prediction, the number of genes in the gut microbiota was 6,767,616 in healthy individuals, 6,166,147 in surviving patients with ICH, and 3,296,140 in deceased patients with ICH, with 3,050,380 shared genes (Figure 1A). The metagenomic results indicated that, at the genus level, the gut microbiota counts were 3,508 in healthy individuals, 3,235 in surviving patients with ICH, and 2,572 in deceased patients with ICH (Figure 1B). Similar trends were observed at the phylum, class, order, and family levels (Supplementary Figure 1). β-diversity analysis indicates distinct compositional differences in gut microbiota between ICH patients and healthy controls. The Binary Jaccard algorithm is used for calculating sample distances (Figure 1C). The α-diversity was determined based on the Simpson index (Figure 1D), Shannon index (Figure 1E), observed species (Figure 1F) indices and Chao1 indices (Supplementary Figure 2). Compared with the healthy control group, species richness and diversity indices were significantly reduced in all ICH groups.

**FIGURE 2:**
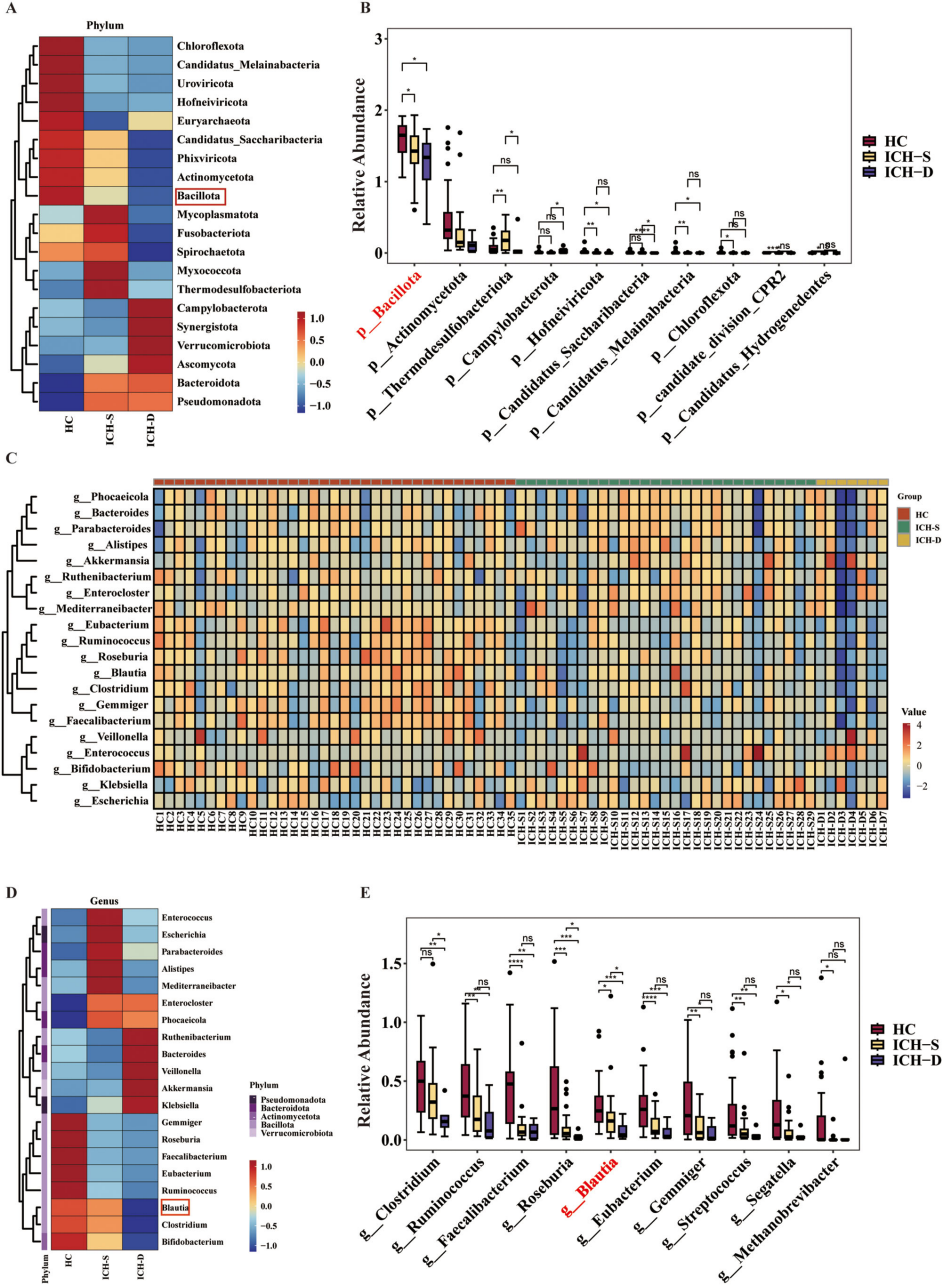
The genus *Blautia* was decreased in the gut of patients with ICH. **(A)** Heatmap of the most differentially abundant features of gut microbiota at the phylum level in the HC, ICH-S, and ICH-D groups. **(B)** Relative abundances of differentially abundant features at the phylum level in the three groups. **(C)** Heatmap of the relative abundance of the gut microbiota at the genus level in the three groups. **(D)** Heatmap of the most differentially abundant features in the gut microbiota of the three groups at the genus level. **(E)** Relative abundances of differentially abundant features at the genus level in the three groups. (*p < 0.05, **p < 0.01, ***p < 0.001 and ****p < 0.0001. HC *n* = 35, ICH-S *n* = 29, ICH-D *n* = 7).

At the phylum level, Bacillota, Actinomycetota, Phixoviricota and candidatus *Saccharibacteria* were notable enriched in the guts of healthy individuals (Figures 2A, B). They also showed some expression in the gut of surviving patients with ICH but were almost non-expressed in the gut of deceased patients with ICH, indicating their potential as probiotic candidates for ICH. At the genus level, *Blautia*, *Clostridium*, and *Bifidobacterium* showed similar trends (Figures 2C, D). Changes in microbial composition at the class, order, and family levels are shown in Supplementary Figure 3. Among them, *Blautia* drew our attention as it was significantly expressed in the HC and ICH-S groups compared to that in the ICH-D group (Figure 2E).

### 3.2 BC ameliorates neurological deficits in ICH mice

**FIGURE 3:**
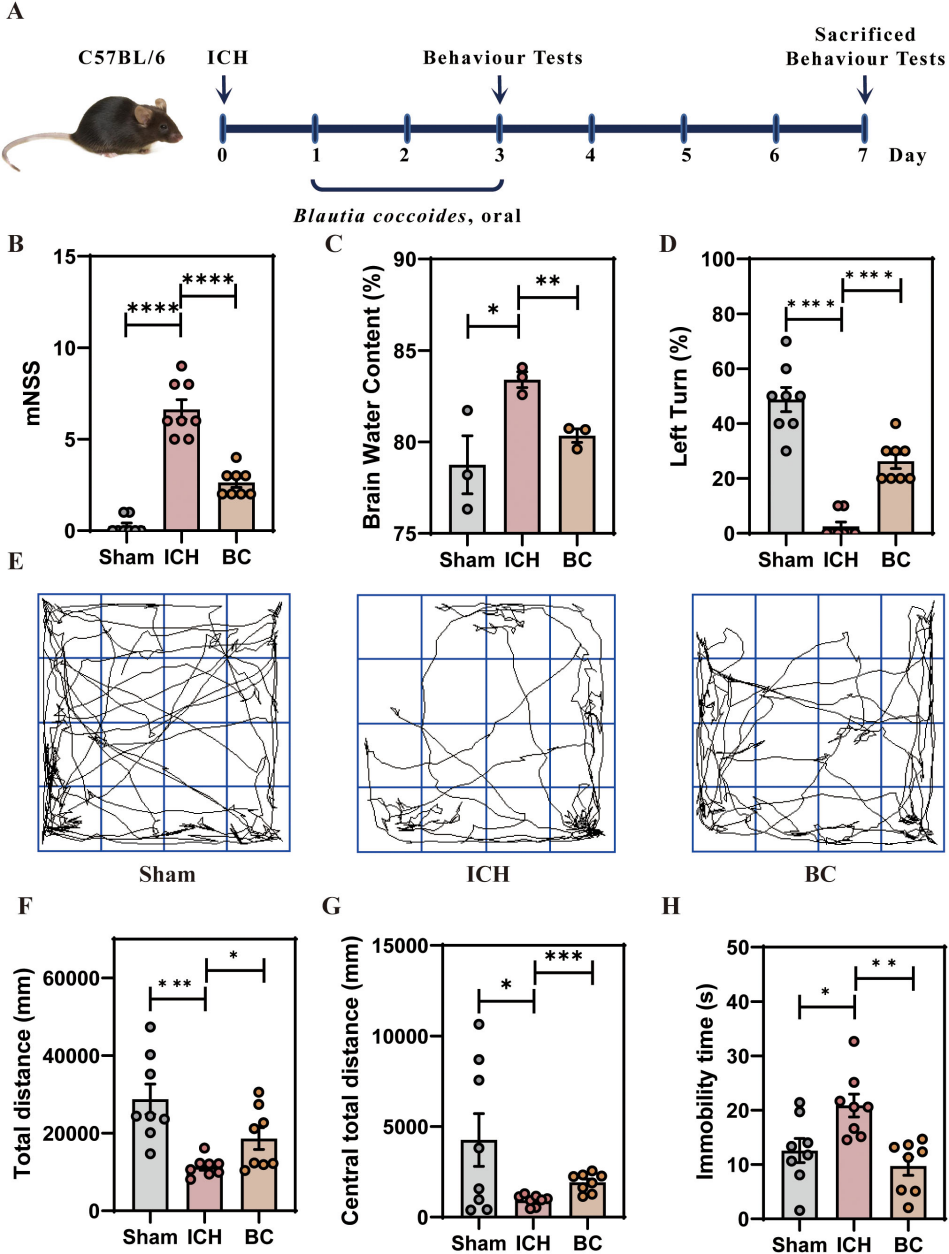
*Blautia coccoides* ameliorated neurological deficits in mice with ICH **(A)** schematic of the experiment. **(B)** Modified neurological severity score (mNSS) (*n* = 8). **(C)** Brain water content (%) (*n* = 3). **(D)** Corner test results (*n* = 8). **(E)** Representative images of the open-field test. **(F)** Total distance moved in the open-field test (*n* = 8). **(G)** Distance moved in the central zone in the open-field test (*n* = 8). **(H)** Immobility time in the open-field test (*n* = 8). (*p < 0.05, **p < 0.01, ***p < 0.001 and ****p < 0.0001).

The experimental design is illustrated in Figure 3A. Beginning on day 1 post-modeling, BC was administered orally to ICH mice for 3 consecutive days. Behavioral changes were assessed at 3 and 7 days post-modeling, respectively. The results showed that, on the third day, there was no significant difference in mNSS scores between the BC and ICH groups (Supplementary Figure 4). However, on the seventh day, the mNSS scores of the BC group were significantly lower than those of the ICH group (Figure 3B). Therefore, the seventh day was chosen as the time point for subsequent assessment. The brain water content in the right cerebral hemisphere tissue was significantly higher in the ICH group than in the sham group (Figure 3C). Brain water content in the BC group was significantly lower than that in the ICH group, indicating that BC intervention can decrease the extent of edema caused by ICH.

Similarly, the corner test indicated that the BC intervention significantly reduced the proportion of right turns after ICH (Figure 3D). The open field test revealed that, compared to those in the sham group, mice in the ICH group exhibited significant motor impairments (Figure 3E), including reduced total distance moved (Figure 3F), decreased distance moved in the central zone (Figure 3G), and slow movement (Figure 3H). BC treatment ameliorated neurological impairment caused by ICH.

### 3.3 BC reduces the histopathological injuries in ICH mice

**FIGURE 4:**
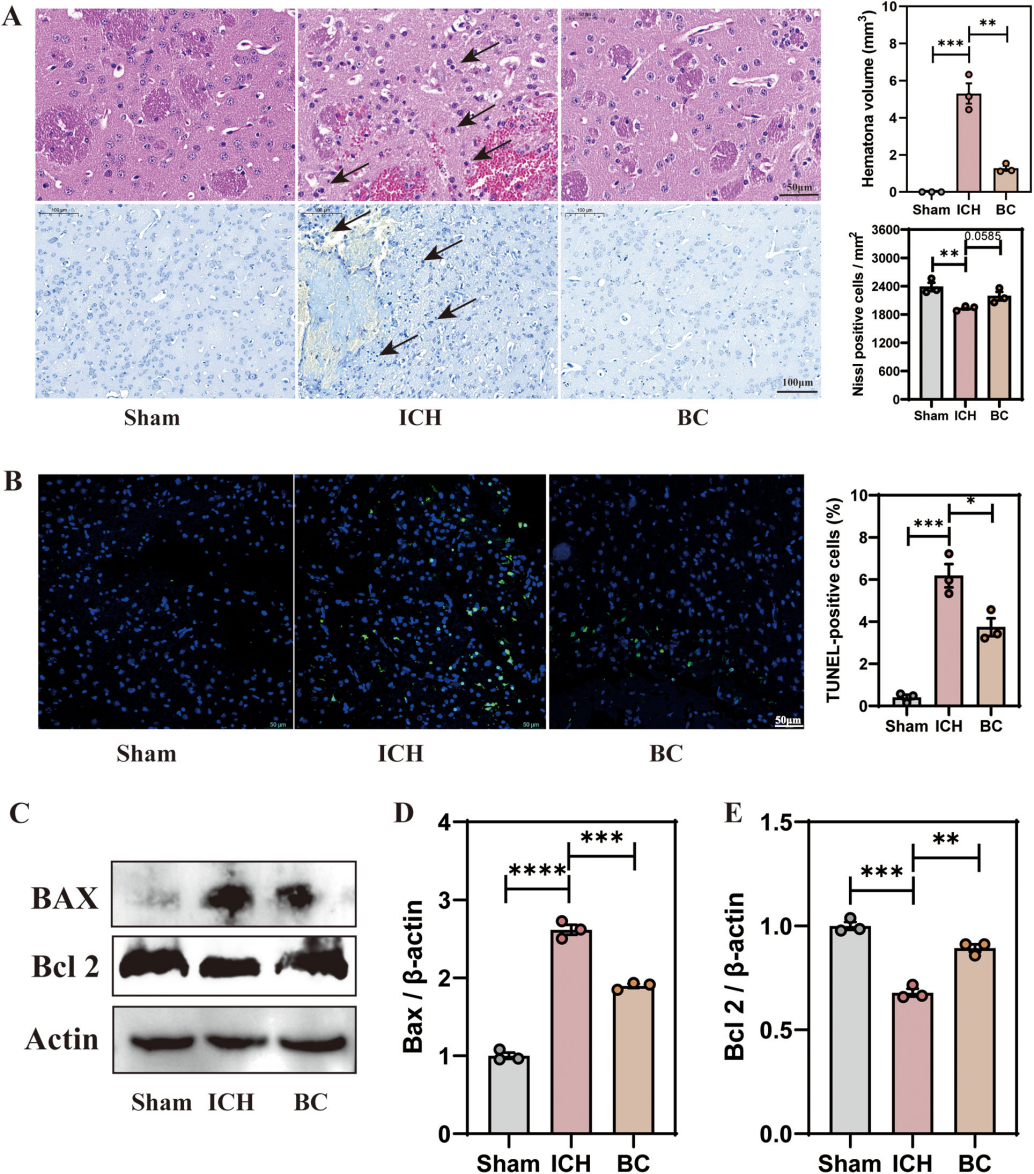
*Blautia coccoides* reduced histopathological injury in mice with ICH **(A)** Representative images of hematoxylin and eosin (H&E) staining (Scale bar: 50 μm) and Nissl staining (Scale bar: 100 μm) in brain sections. **(B)** Representative images of TdT-mediated dUTP nick-end labeling (TUNEL) staining. Scale bar: 50 μm. **(C)** Representative western blot images of Bax and Bcl 2. **(D)** Quantitative densitometric analysis of Bax expression. **(E)** Quantitative densitometric ratio of Bcl 2. (*p < 0.05, **p < 0.01, ***p < 0.001 and ****p < 0.0001. *n* = 3).

To further investigate the changes in brain tissue after ICH induction, we assessed the level of neuronal damage 7 days post-ICH induction. HE and Nissl staining indicated that the neuronal cells in the sham group exhibited normal morphology and size, with a dense and uniform arrangement and clear layers. In the ICH group, damaged neuronal cells around the hematoma exhibited neuronal cell shrinkage and deformation, nuclear condensation, nuclear fragmentation, and decreased Nissl bodies. BC treatment increased the number of surviving cells with clear neuronal cell edges, increased the number of Nissl bodies, and restored morphology (Figure 4A).

TUNEL staining revealed the presence of TUNEL-positive cells in the cerebral cortex, which were almost absent in the sham group (*p* < 0.001) (Figure 4B). The number of TUNEL-positive cells was lower in the BC treatment group than in the ICH group. Finally, we assessed the expression levels of apoptosis-associated proteins in the mouse cerebral cortex by western blot analysis (Figure 4C). The results showed that, compared with the sham group, the expression level of the apoptotic regulator Bax was significantly increased in the ICH group, whereas Bax was significantly decreased in the BC group (Figure 4D). In comparison with the sham group, the expression level of B-cell lymphoma 2 (Bcl 2) in the cerebral cortex of ICH mice was significantly decreased, and the BC treatment group showed significantly increased expression level of Bcl 2 (Figure 4E). BC treatment significantly alleviated the histological damage and neuronal apoptosis following ICH induction.

### 3.4 BC alleviates neuroinflammation in ICH mice

**FIGURE 5:**
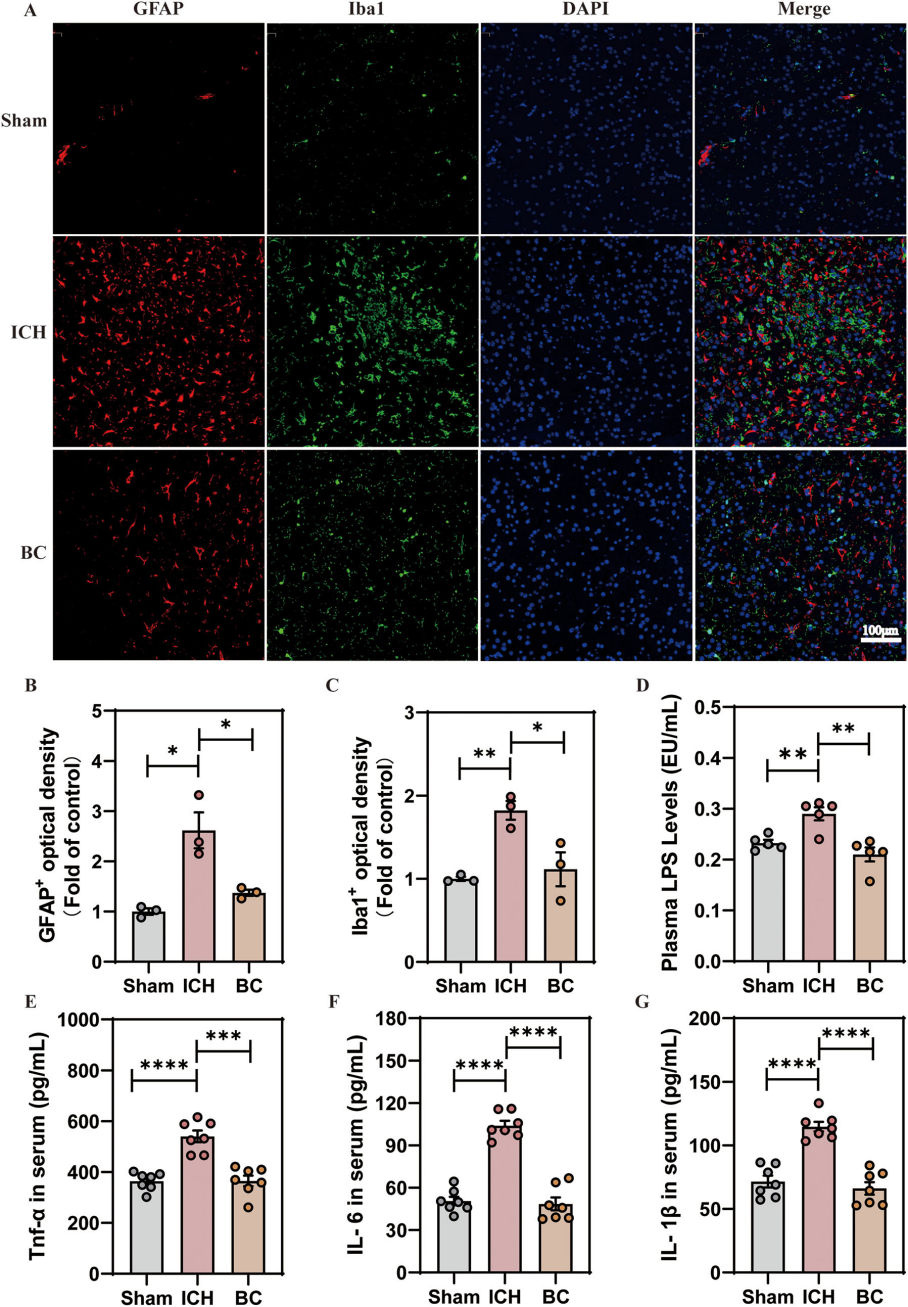
**(A)** Representative images of immunofluorescence staining for glial fibrillary acidic protein (GFAP; red) and Iba1 (green) (*n* = 3). **(B)** Relative GFAP fluorescence intensity (*n* = 3). **(C)** Relative fluorescence intensity of Iba1 (*n* = 3). **(D)** Plasma LPS levels (*n* = 6). **(E)** Serum TNF-α levels were determined by ELISA (*n* = 6). **(F)** Serum IL-6 levels were determined by ELISA (*n* = 6). **(G)** Serum IL-β levels were determined by ELISA (*n* = 6). (*p < 0.05, **p < 0.01, ***p < 0.001 and ****p < 0.0001. *n* = 3).

Neuroinflammation is a key factor in the development of secondary brain damage following ICH (Guo et al., 2023). Extensive activation of glial cells promotes the release of pro-inflammatory factors and infiltration of peripheral immune cells (Wang et al., 2022). Therefore, we assessed the levels of glial cell activation in the mouse brain and the expression levels of pro-inflammatory factors in the serum following ICH. We selected GFAP as a marker for astrocytes and Iba1 as a marker for microglia. The results indicated a significant increase in the expression levels of GFAP and Iba1 in the cerebral cortex of mice with ICH, suggesting a significant activation of astrocytes and microglia. In contrast, the expression levels of GFAP and Iba1 in the cerebral cortex of ICH mice treated with BC significantly decreased, indicating that BC treatment reduced the activation of astrocytes and microglia (Figures 5A–C).

We assessed the expression levels of LPS and pro-inflammatory cytokines (TNF-α, IL-6, and IL-1β) in the serum (Figures 5D–G). The results showed a significant increase in the expression levels of LPS, TNF-α, IL-6, and IL-1β in mice with ICH, whereas the expression levels of LPS, TNF-α, IL-6, and IL-1β in the serum of ICH mice after BC intervention significantly decreased. BC alleviated neuroinflammation in mice following the ICH induction.

### 3.5 BC regulates the composition of gut microbiota in ICH mice

**FIGURE 6:**
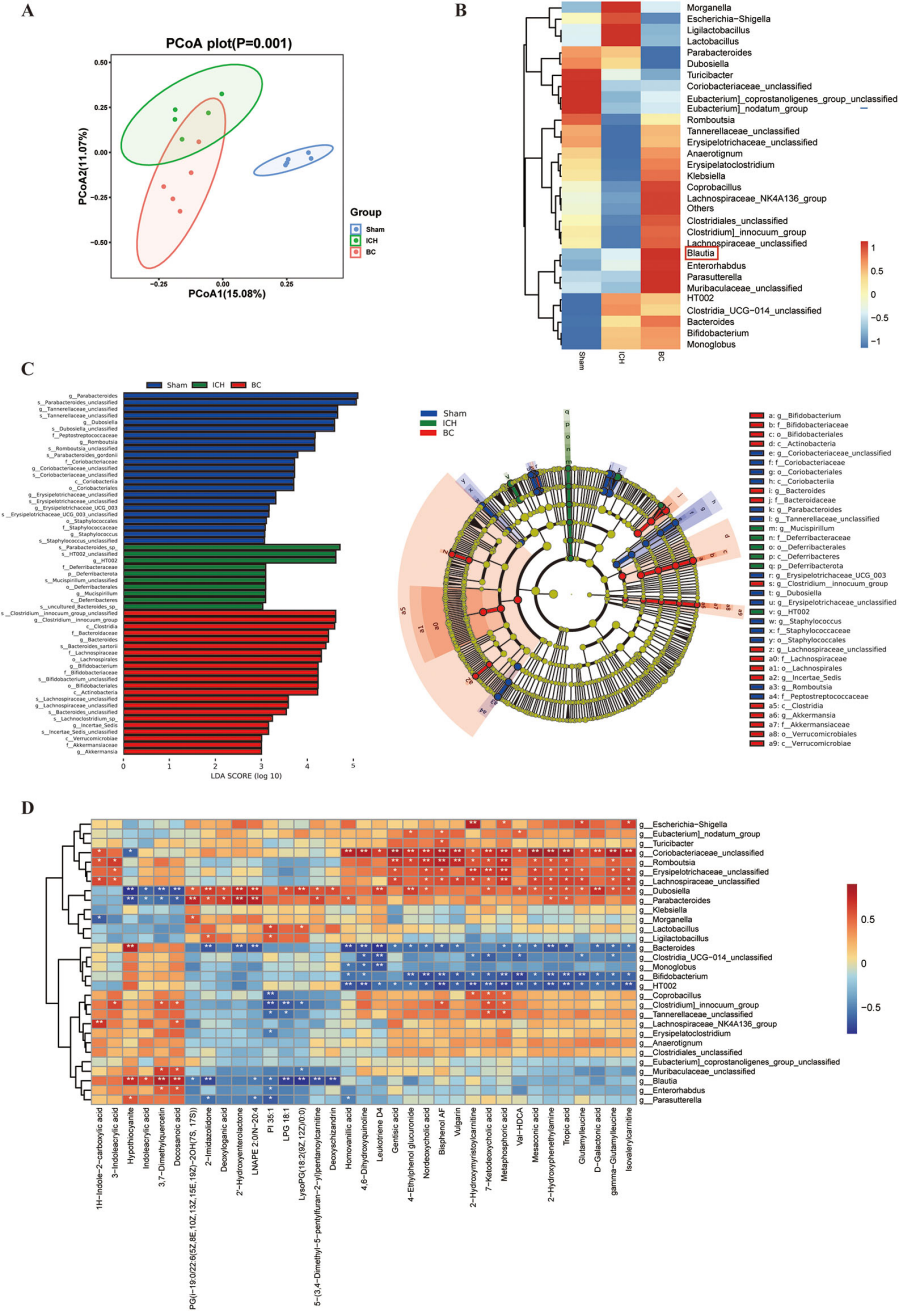
*Blautia coccoides* regulates the composition of gut microbiota in ICH mice. **(A)** The β-diversity of the gut microbiota was represented by PCoA analysis in the sham, ICH, and BC groups. **(B)** Heatmap of the relative abundance of the gut microbiota at the genus level in the three groups. **(C)** Linear discriminant analysis (LDA) score histograms of the differential bacteria. **(D)** Spearman correlation heatmap of the gut microbiota and serum metabolites (*n* = 5).

To determine the colonization levels of BC in the intestines of ICH mice, we conducted 16S rRNA sequencing to analyze the gut microbial composition of ICH mice treated with BC. β-Diversity analysis revealed distinct clustering of microbial compositions among the sham, ICH, and BC groups (Figure 6A). The heatmap indicated that the BC group had the highest levels of BC in the intestine, demonstrating successful colonization (Figure 6B). In addition, BC restored the intestinal microbial dysbiosis caused by ICH. *Romboutsia*, *Erysipelotrichaceae*, *Anaerotignum*, *Erysipelatoclostridium*, *Klebsiella*, *Coprobacillus*, *Lachnospiraceae*, *Clostridiales*, *Clostridium*, and *Lachnospiraceae* were significantly decreased in the intestines of ICH mice, but significantly increased in the intestines of ICH mice with BC intervention. Conversely, *Morganella*, *Escherichia-Shigella*, *Ligilactobacillus*, and *Lactobacillus* were present at higher levels in the intestines of ICH mice, whereas their levels were significantly lower in the intestines of mice in the sham and BC groups. The (LDA) effect further explains these results (Figure 6C). In addition, we conducted a serum metabolite analysis of ICH mice treated with BC. These results indicate that the therapeutic effect of BC in ICH mice may be associated with metabolites, such as hypothiocyanite, indoleacrylic acid, 3,7-dimethylquercetin, docosanoic acid, 2-imidazolidone, and deoxyschizandrin (Figure 6D). Functional enrichment analysis of dysregulated gut microbiota indicates that BC ameliorates ICH potentially through modulating lipid metabolism pathways (Supplementary Figure 5).

## 4 Discussion

Given the increasing number of studies reporting the significant role of gut microbiota dysbiosis in the pathogenesis of ICH, we conducted a metagenomic analysis to investigate changes in the gut microbiota of healthy individuals, surviving patients with ICH, and deceased patients with ICH. This study aimed to evaluate the role of gut microbiota in the development of ICH and to screen representative strains to assess their therapeutic effects on ICH. Our findings suggest that *Blautia* may serve as a key bacterial biomarker for intervention in ICH. In addition, we established a mouse model of ICH using an autologous stereotactic injection. BC effectively ameliorated secondary damage in ICH mice, including neurological functional impairment, histological damage, neuroinflammation, and the restoration of intestinal homeostasis.

Gut dysbiosis is important in the pathogenesis of neurological disorders. Several studies have reported dysbiosis of gut microbiota in patients with ICH (Wang et al., 2024; Luo et al., 2022; Li et al., 2022). Furthermore, the reconstitution of healthy gut microbiota in ICH mice has been shown to improve functional deficits and neuroinflammation (Li et al., 2023; Sun and Xu, 2023; Yu et al., 2021a). In addition, cell tracking studies have demonstrated the migration of gut lymphocytes to the brain following ICH (Yu et al., 2021b). These findings suggest that gut microbiota substantially impacts ICH outcomes. Consistent with previous research, there was a decrease in the diversity and abundance of the gut microbiota in patients with ICH. The most abundant phylum were Bacillota and Actinomycetota (Wang et al., 2024). Furthermore, at the family level, *Lachnospiraceae* exhibited the highest abundance and was significantly reduced in the intestines of patients with ICH (Wang et al., 2024). At the genus level, *Faecalibacterium* was significantly decreased in the intestines of patients with ICH, whereas *Enterococcus* was significantly enriched in the intestines of surviving patients with ICH (Luo et al., 2022). Multiple studies have identified *Enterococcus* as a biomarker of ICH in multiple studies (Wang et al., 2024; Luo et al., 2022). In our study, *Enterococcus* was significantly enriched in the intestines of surviving patients with ICH, but was not expressed in the intestines of deceased patients with ICH. However, the potential reasons for this discrepancy require further investigation. Consistent with these findings, our results demonstrated significant enrichment of the *Roseburia* and *Blautia* genus in both healthy controls and surviving ICH patients, suggesting their potential role as keystone taxa influencing ICH prognosis. This observation warrants further mechanistic investigation.

Recent studies have provided evidence for the neuroprotective effects of certain *Blautia* species. *Blautia* species are significantly decreased in the gut of patients with various neurological disorders, including Parkinson’s disease, Alzheimer’s disease, anorexia nervosa, multiple sclerosis, adolescent depression, and severe depression (Guo et al., 2022; Marizzoni et al., 2023; Prochazkova et al., 2021; Cox et al., 2021; Zhou et al., 2023; Yang et al., 2020). Specific subspecies of genus *Blautia* have been reported to exhibit significant neuroprotective effects. For example, *Blautia stercoris* MRx0006 alleviates social deficits, repetitive behaviors, and anxiety-like behaviors in a mouse model of autism (Sen et al., 2022). Administration of *Blautia producta* improved the loss of dopaminergic neurons, alleviated motor dysfunction, and inhibited microglia-mediated neuroinflammation in a mouse model of Parkinson’s (Liu J. et al., 2024). BC, a representative strain of *Blautia*, has been reported to exert significant beneficial effects on its hosts. BC improves metabolic disorders and enhances the host’s innate immune response to systemic infections by intestinal and influenza viruses while maintaining intestinal mucus levels (Niu et al., 2024; Wang et al., 2023; Qiu et al., 2024; Holmberg et al., 2024). However, there are no reports of BC in the context of the ICH. In our experiments, we verified that BC is significantly decreased in the gut of patients with ICH, and BC exhibits significant protective effects in ICH mice created through autologous blood injection into the brain. These findings suggest a potential role of BC in the modulation of neurological disorders, particularly ICH. Further research is required to elucidate the specific mechanisms underlying the neuroprotective effects of BC and its potential therapeutic application in neurological disorders. Nonetheless, the observed effects of *Blautia* species and BC highlight the importance of the gut microbiota in neurological health and provide a potential avenue for developing novel interventions targeting the gut-brain axis.

Secondary injury is an important factor contributing to poor prognosis in patients with ICH. The potential mechanisms underlying secondary injury include: (1) Red blood cell lysis, thrombin-induced edema, and neuronal damage, which can lead to significant brain edema and inflammatory cell infiltration due to the release of red blood cell components and thrombin (Tseng et al., 2023). In our experimental results, brain water content increased significantly, and inflammatory cell leakage was observed. (2) Toxic reactions and oxidative damage caused by toxic substances: Toxic substances released during ICH can induce toxic reactions and oxidative damage, leading to neuronal apoptosis (Ren et al., 2022). Consistent with our experimental results, the proportion of apoptotic cells in the cerebral cortex of the ICH mice was significantly increased. (3) Inflammatory response: The inflammatory response is a crucial pathway for secondary brain injury following ICH. Glial cell activation, which is mainly attributed to the release of cytokines and other immune-active molecules, plays a key role in the development of secondary injuries (Ohashi et al., 2023). In our study, astrocytes and microglia in the cerebral cortex of ICH mice were significantly activated, and the expression levels of pro-inflammatory factors, such as LPS, TNF-α, IL-6, and IL-1b in the serum were also significantly elevated. These results indicate the successful modeling of ICH in our mice. Similarly, our study suggests that BC can significantly inhibit the secondary damage caused by ICH. BC-mediated amelioration of ICH appears mechanistically linked to elevated production of four anti-inflammatory metabolites: hypothiocyanite (antimicrobial effector), indoleacrylic acid, 3′,7-dimethylquercetin, and docosanoic acid, all documented to mitigate neuroinflammation (Meredith et al., 2022; Wlodarska et al., 2017; Park et al., 2022; Liu K. et al., 2024).

This study has several limitations that warrant consideration. First, the limited availability of clinical specimens constrained our sample size, potentially reducing the statistical power of subgroup analyses. To address this, efforts to expand the cohort are currently underway to enhance both statistical power and generalizability. Second, our neurobehavioral assessment was restricted to motor and anxiety-like behaviors, as evaluated by the open field test, and did not include higher cognitive domains typically assessed by the Morris water maze. This omission is due to the specialized equipment required for such assessments. Future studies should incorporate hippocampal-dependent spatial memory testing to fully characterize cognitive sequelae following ICH. Third, although metabolites such as short-chain fatty acids are plausible mediators of the effects of bacterial communities, we did not conduct rescue experiments with exogenous supplementation. This mechanistic validation is planned for future investigations.

## 5 Conclusions

Patients with ICH exhibit decreased richness and diversity of gut microbiota. The genus *Blautia*, which is highly expressed in the intestines of healthy individuals, also showed elevated expression in the intestinal tracts of surviving patients with ICH, whereas it was nearly absent in the intestines of deceased patients with ICH. This suggests that *Blautia* is a potential probiotic for patients with ICH. *In vivo* experiments in mice have shown that BC alleviates neurological functional damage caused by ICH. Furthermore, BC significantly inhibited histological damage, neuroinflammation, and intestinal dysfunction induced by ICH. This study explores the role of the genus *Blautia* in the intestines of patients with ICH and validates, at the animal level, the mechanisms by which BC alleviates secondary damage in ICH mice. To our knowledge, this study is the first to elucidate the therapeutic effects of BC on brain injury.

## Data Availability

The raw sequence data reported in this manuscript have been deposited in the Genome Sequence Archive (Genomics, Proteomics & Bioinformatics 2021) in National Genomics Data Center (Nucleic Acids Res 2022), China National Center for Bioinformation/Beijing Institute of Genomics, Chinese Academy of Sciences (GSA: CRA028361) that are publicly accessible at https://ngdc.cncb.ac.cn/gsa.

## Supplementary material

Supplementary Figure 1 **(A)** Venn diagram of gut microbiota counts at the phylum level in the HC, ICH-S, and ICH-D groups. **(B)** Venn diagram of the gut microbiota counts at the class level in the three groups. **(C)** Venn diagram of the gut microbiota counts at the order level in the three groups. **(D)** Venn diagram of the gut microbiota counts at the family level in the three groups. (HC *n* = 35, ICH-S *n* = 29, ICH-D *n* = 7).

Supplementary Figure 2 The α-diversity of gut microbiota is represented by chao1 index in the three groups. (**p* < 0.05, HC *n* = 35, ICH-S *n* = 29, ICH-D *n* = 7).

Supplementary Figure 3 **(A)** Heatmap analysis of the relative abundance of the gut microbiota at the class level in HC, ICH-S, and ICH-D groups. **(B)** Heatmap analysis of the relative abundance of the gut microbiota at the order level in the three groups. **(C)** Heatmap analysis of the relative abundance of the gut microbiota at the family level in the three groups. (**p* < 0.05, ***p* < 0.01, and ****p* < 0.001. HC *n* = 35, ICH-S *n* = 29, ICH-D *n* = 7).

Supplementary Figure 4 Modified neurological severity score (mNSS) at 3 days after ICH modeling (*n* = 8).

Supplementary Figure 5 Functional enrichment analysis of dysregulated gut microbiota in BC-treated ICH mice.
