# Supplementary material for: The protective effect of Blautia coccoides in secondary injury of intracerebral hemorrhage
Source: Front Microbiol. 2025 Sep 2;16:1616222. doi: 10.3389/fmicb.2025.1616222 (PMC12436306; doi:10.3389/fmicb.2025.1616222)
Supplement: Supplementary file 1 [file Table_1.doc]

Supplementary Table 1: Demographic and Clinical Characteristics of Study Participants

| Groups | Variable | Healthy | ICH-S | ICH-D |
| --- | --- | --- | --- | --- |
| SEX | Male | 22 | 16 | 5 |
|  | Female | 13 | 13 | 2 |
| Age | Average age | 61.14±10.84 | 62.69±13.23 | 58.57±16.95 |
|  | Median age | 60 | 68 | 51 |
| Admitting diagnosis |  | _ | Intracerebral hemorrhage | Intracerebral hemorrhage |
| Sampling Time^a^ | Average Time | _ | 8.58±3.43 | 9.57±3.19 |
|  | Median Time | _ | 9 | 10 |
| Place of origin | Local residents^b^ | 31 | 21 | 5 |
|  | Other regions | 4 | 8 | 2 |

Note: ^a^Time from hospital admission to sample collection (days);

^b^ Local residents defined as born and residing in Zhejiang Province for ≥10 years;
