# Supplementary figures and images for: The protective effect of Blautia coccoides in secondary injury of intracerebral hemorrhage
Source: Front Microbiol. 2025 Sep 2;16:1616222. doi: 10.3389/fmicb.2025.1616222 (PMC12436306; doi:10.3389/fmicb.2025.1616222)

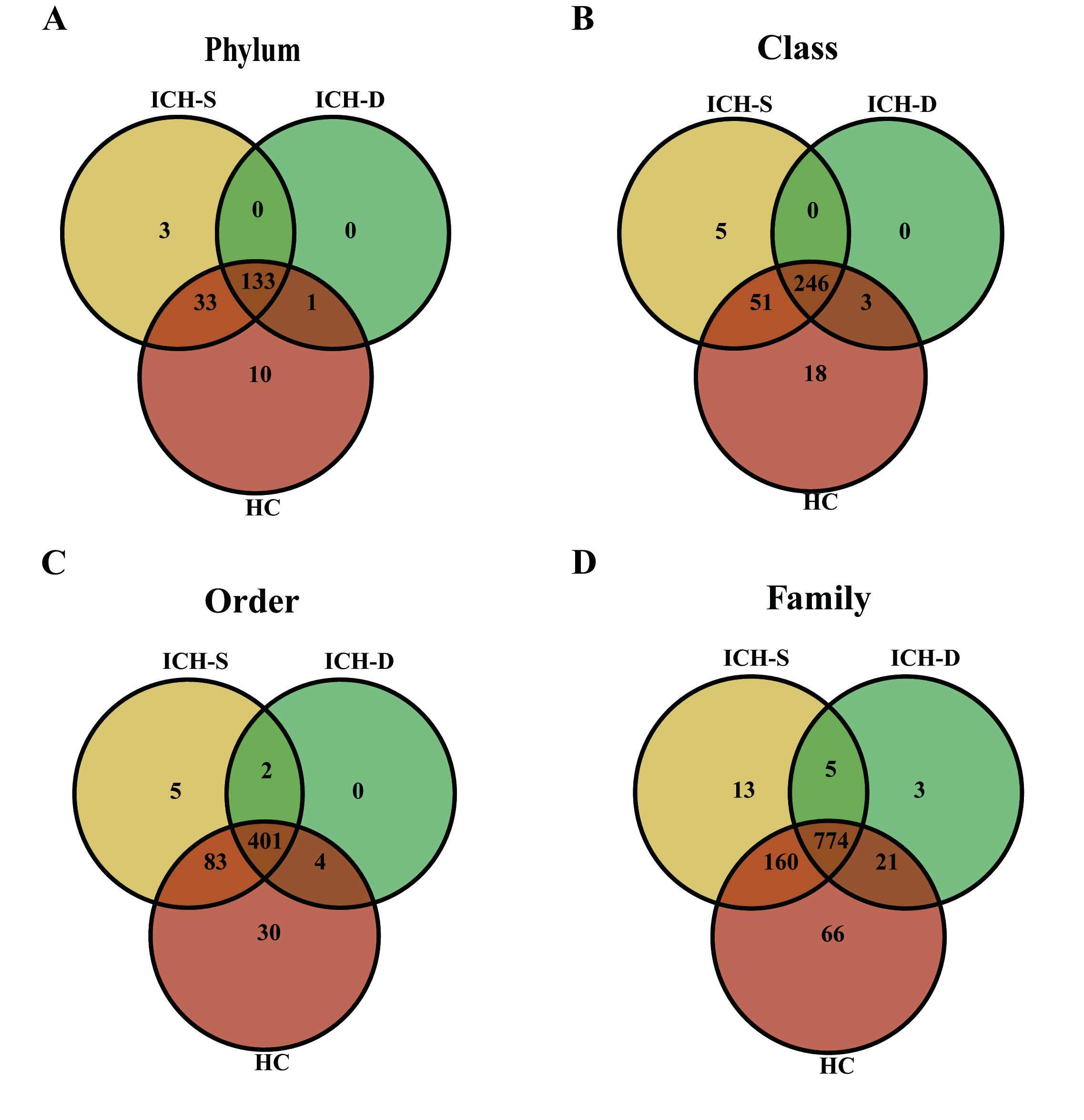

Supplement: Supplementary Figure 1 — (A) Venn diagram of gut microbiota counts at the phylum level in the HC, ICH-S, and ICH-D groups. (B) Venn diagram of the gut microbiota counts at the class level in the three groups. (C) Venn diagram of the gut microbiota counts at the order level in the three groups. (D) Venn diagram of the gut microbiota counts at the family level in the three groups. (HC n = 35, ICH-S n = 29, ICH-D n = 7). [file Image_1.tif]

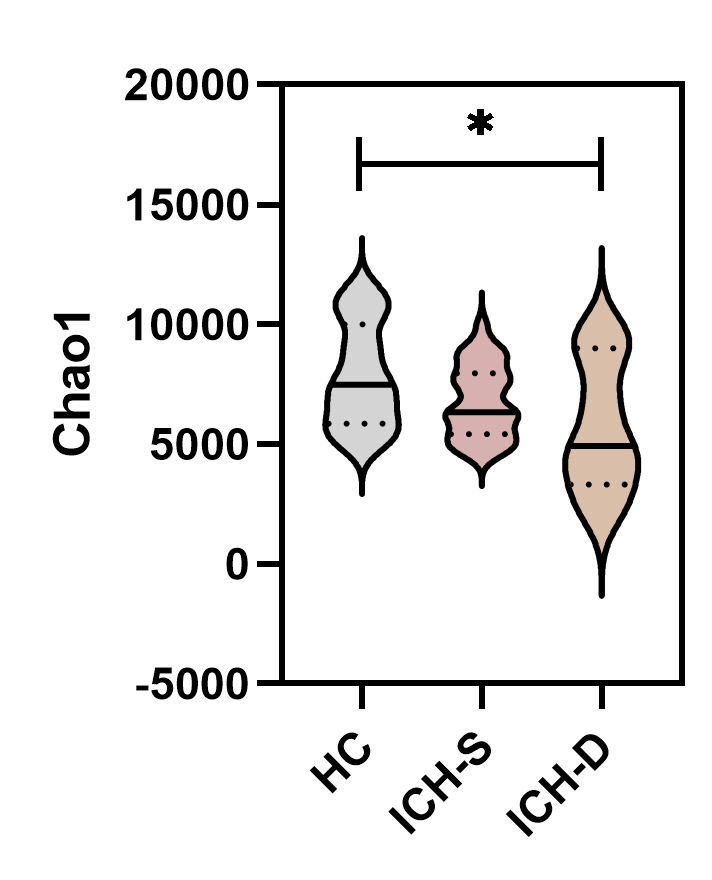

Supplement: Supplementary Figure 2 — The α-diversity of gut microbiota is represented by chao1 index in the three groups. (*p < 0.05, HC n = 35, ICH-S n = 29, ICH-D n = 7). [file Image_2.tif]

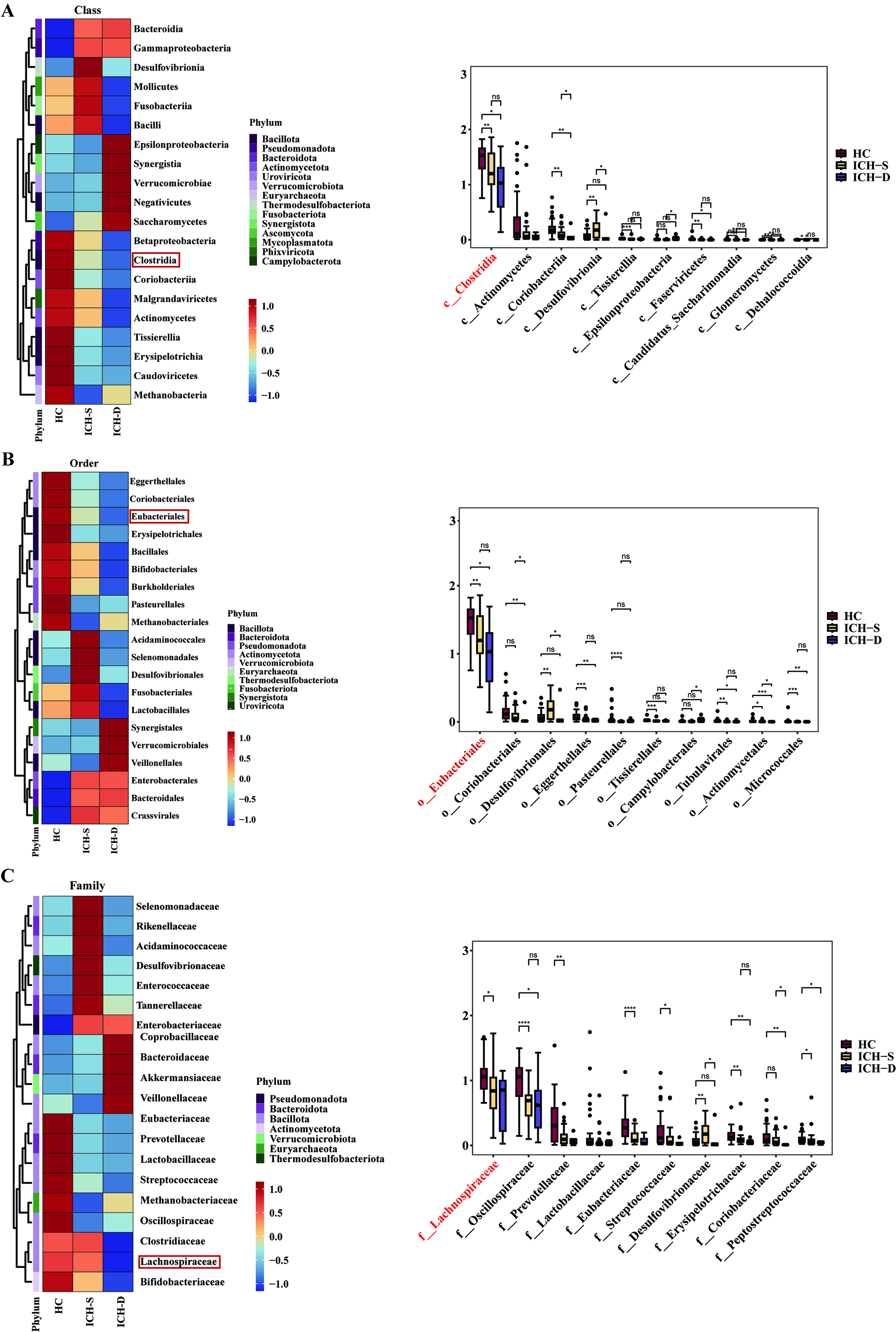

Supplement: Supplementary Figure 3 — (A) Heatmap analysis of the relative abundance of the gut microbiota at the class level in HC, ICH-S, and ICH-D groups. (B) Heatmap analysis of the relative abundance of the gut microbiota at the order level in the three groups. (C) Heatmap analysis of the relative abundance of the gut microbiota at the family level in the three groups. (*p < 0.05, **p < 0.01, and ***p < 0.001. HC n = 35, ICH-S n = 29, ICH-D n = 7). [file Image_3.tif]

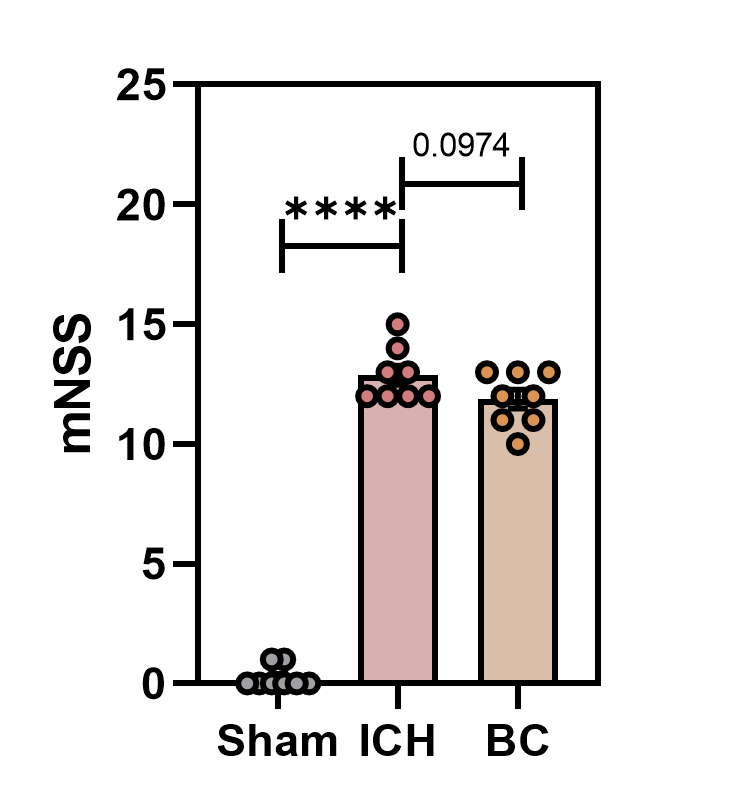

Supplement: Supplementary Figure 4 — Modified neurological severity score (mNSS) at 3 days after ICH modeling (n = 8). [file Image_4.tif]

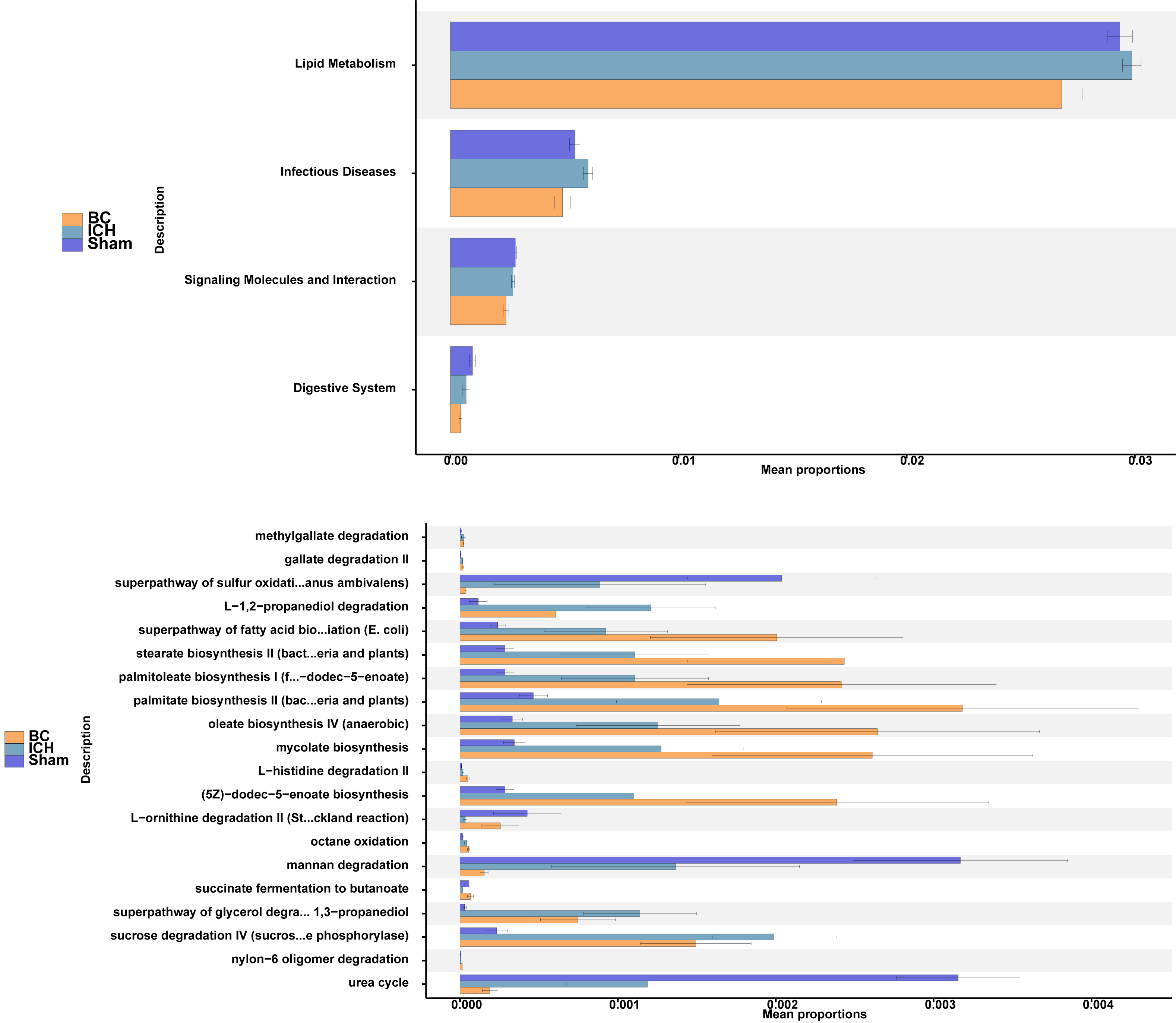

Supplement: Supplementary Figure 5 — Functional enrichment analysis of dysregulated gut microbiota in BC-treated ICH mice. [file Image_5.tif]
